# Chitosan Loaded into a Hydrogel Delivery System as a Strategy to Treat Vaginal Co-Infection

**DOI:** 10.3390/pharmaceutics10010023

**Published:** 2018-02-03

**Authors:** Diego R. Perinelli, Raffaella Campana, Athanasios Skouras, Giulia Bonacucina, Marco Cespi, Francesca Mastrotto, Wally Baffone, Luca Casettari

**Affiliations:** 1 School of Pharmacy, University of Camerino, Via Gentile III da Varano, 62032 Camerino (MC), Italy; diego.perinelli@unicam.it (D.R.P.); giulia.bonacucina@unicam.it (G.B.); marco.cespi@unicam.it (M.C.); 2 Department of Biomolecular Sciences, University of Urbino, Piazza del Rinascimento n° 6, 61029 Urbino (PU), Italy; raffaella.campana@uniurb.it (R.C.); nasosskouras@upatras.gr (A.S.); wally.baffone@uniurb.it (W.B.); 3 Department of Pharmaceutical and Pharmacological Sciences, University of Padova, Via F. Marzolo n° 5, 35131 Padova (PD), Italy; francesca.mastrotto@unipd.it

**Keywords:** mixed hydrogels, nanoparticles, Minimum Inhibitory Concentration (MIC), mucoadhesiveness, *Candida* spp.

## Abstract

Polymeric hydrogels are common dosage forms designed for the topical administration of antimicrobial drugs to treat vaginal infections. One of the major advantages of using chitosan in these formulations is related to the intrinsic and broad antimicrobial activity exerted on bacteria and fungi by this natural polymer. Most vaginal yeast infections are caused by the pathogenic fungus *Candida albicans*. However, despite the anti-Candida activity towards and strains susceptibility to low molecular weight chitosan being documented, no information is available regarding the antimicrobial efficacy of mixed hydrogels in which chitosan is dispersed in a polymeric matrix. Therefore, the aim of the study is to evaluate the anti-Candida activity against eight different albicans and non-albicans strains of a mixed hydroxypropyl methylcellulose (HPMC)/chitosan hydrogel. Importantly, chitosan was dispersed in HPMC matrix either assembled in nanoparticles or in a monomolecular state to eventually correlate any variation in terms of rheological and mucoadhesive properties, as well as anti-Candida activity, with the chitosan form. Hydrogels containing 1% *w*/*w* chitosan, either as free polymer chain or assembled in nanoparticles, showed an improved mucoadhesiveness and an anti-Candida effect against all tested albicans and non-albicans strains. Overall, the results demonstrate the feasibility of preparing HPMC/CS mixed hydrogels intended for the prevention and treatment of Candida infections after vaginal administration.

## 1. Introduction

Chitosan has been extensively exploited to develop topical formulations for ocular, mucosal or skin applications thanks to its mucoadhesiveness and antimicrobial activity [1]. Aimed at prolonging the residential time of the drug inside the vaginal cavity and to enhance the antibacterial or antifungal activity of the delivered drug, several vaginal dosage forms containing chitosan have been developed as films, tablets, inserts, hydrogels, and more recently, liposomes [2,3,4,5,6,7]. Among all these formulations, hydrogels are still the most used, allowing easy and precise administration and being largely versatile [8]. Moreover, hydrogels represent a non-expensive platform and they additionally exert a moisturizing/lubricant effect on the mucosa [9]. Several mucoadhesive polymers have been investigated for the preparation of hydrogels intended for vaginal applications. Among those, cellulose derivatives, carboxypolymethylene and chitosan have been particularly investigated to improve the contact of the formulation with the vaginal mucosa and to reduce the dilution and clearance of the hydrogels by the vaginal fluids [10].

For instance, Bonferoni et al. reported the preparation of a 3% chitosan-based hydrogel containing lactic acid for the maintenance of the physiological vaginal pH [11]. In recent years, the field of microbiocide drug delivery to the vaginal cavity has been developing a nanomedicine-based approach using hydrogel embedded polymeric nanoparticles for the treatment or prevention of vaginal infections [12,13]. Polymeric nanoparticles have been successfully incorporated in chitosan-based hydrogels for vaginal administration in order to promote the drug penetration inside the mucosa [14,15]. On the other side, chitosan nanoparticles were also dispersed in polymeric hydrogels intended for different routes of drug administration [16,17]. The variety of chitosan nanoparticle possible applications is closely related to their well-documented antibacterial activity [18]. Many marketed hydrogels, intended for the treatment of vaginosis, contain metronidazole and are effective against bacterial infections caused by vaginal bacteria and protozoa, such as *Gardnerella Vaginalis* and *Trichomonas Vaginalis*. However, they are ineffective or display a low activity against the common yeast co-infection by *Candida* spp. Indeed, a common therapeutic approach for *Candida* spp. is topical treatment with azole antifungal drugs (e.g., clotrimazole), formulated as creams (due to their low solubility in water). In this scenario, the antimycotic activity of chitosan has been well documented, despite some controversial data being reported in regard to the major effect of nanoparticles when compared to chitosan as free polymer [19,20]. As such, chitosan nanoparticles could be employed to broaden the antimicrobial activity of drug-loaded vaginal hydrogel formulations toward *Candida* spp. infections.

We propose here a formulation of a mixed hydrogel based on chitosan (in solution or as nanoparticles) dispersed in a hydroxypropyl methylcellulose (HPMC) matrix loaded with metronidazole (MTZ) (as an antimicrobial drug) for vaginal application. The main goal of the work was to assess if chitosan could be successfully employed to improve the physico-chemical properties of polymeric hydrogels loaded with metronidazole, while broadening their spectrum of antimicrobial activity, including *Candida* co-infections. To this aim, we investigated the effect of chitosan, either as nanoparticles or as free polymer, on the rheological, mucoadhesive properties and anti-Candida activity of these hydrogel preparations.

## 2. Materials and Methods 

### 2.1. Materials

Chitosan (CS) ChitoClear^®^ (viscosity 9 Cp, Mw < 50 kDa, 90% degree of deacetylation, DD) was kindly supplied by Primex (Siglufjordur, Iceland). Hydroxypropylmethyl cellulose (HPMC) K4M Premium EP was supplied by Colorcon (Milan, Italy). Carboxypolymethylene polymer (Carbopol 974P) was kindly supplied by Lubrizol (IMCD, Barcelona, Spain). Sodium tripolyphosphate (TPP) and mucin from porcine stomach type II were purchased from Sigma–Aldrich (Milan, Italy). Metronidazole (MTZ) was purchased from ACEF (Fiorenzuola d'Arda, Italy). Ultrapure water (18.2 MΩ·cm) was obtained by reverse osmosis using a Direct-Q^®^ 3 UV (Millipore, Milan, Italy) apparatus.

### 2.2. Bacterial Strains and Culture Conditions

Eight strains of *Candida* spp., kindly furnished by Gamma Laboratory (Pesaro, Italy), were used in this study: *C. albicans* 11/01, *C. albicans* 18/01, *C. albicans* 4940, *C. albicans* 360923, *C. glabrata* 104/1, *C. glabrata* 104/22, *C. glabrata* 4955 and *C. lusitaniae* 360804. All the strains were isolated from urinary human specimens (vaginal swabs) and identified by chromogenic Candida agar (CCA, Biolife, Milan, Italy). The strains were routinely grown on Sabouraud Dextrose Agar (SDA) (Liofilchem, Roseto degli Abruzzi, Italy) plates, incubated at 37° C for 24 h. Stock cultures were maintained at −80 °C in nutrient broth (Oxoid, Milan, Italy) with 15% of glycerol.

### 2.3. Methods

#### 2.3.1. Preparation of Chitosan Nanoparticles (NPs)

Chitosan (CS) nanoparticles were obtained by a slightly modified gelation procedure, first reported by Calvo et al. [21]. A 2% *w*/*w* stock dispersion of chitosan was prepared in acetate buffer, pH 4.5 [22], and left stirring at room temperature for 24 hr. Nanoparticles were prepared at three chitosan concentrations (0.1%, 0.5% and 1% *w*/*w*) and using different CS/TPP ratios. Briefly, 5 mL of tripolyphosphate solution (containing the adequate amount of TPP) in acetate buffer, pH 4.5, was added dropwise (at a rate of 200 μL/min) under stirring to 5 mL of CS dispersion in acetate buffer, pH 4.5, at a double concentration as compared to that in the final preparation. CS nanoparticles were prepared at different CS/TPP ratios according to CS concentrations. For 0.1% *w*/*w* CS final concentration, nanoparticles were prepared at a CS/TPP weight ratios between 1:1 to 12:1, while for 0.5% and 1% *w*/*w* CS concentration, nanoparticles were prepared at ratios ranging from 2:1 to 18:1.

#### 2.3.2. Characterization of Chitosan Nanoparticles

CS nanoparticles were characterized by dynamic light scattering (DLS) and transmittance analysis. The hydrodynamic diameters (nm) and the scattering intensity (counts, KCps) were measured by DLS using a Zetasizer Nano S instrument (Malvern Instruments, Ltd., Malvern, UK). Briefly, 1 mL of the sample was placed in a disposable cuvette and analysed at 25 °C, after 180 s of equilibration time. The transmittance was measured at 660 nm using a UV-1800 spectrophotometer (Shimadzu, Milan, Italy) at 25 °C. Transmittance of nanoparticles was reported as a relative percentage (%) with the respect to that measured for the dispersion containing chitosan at the same concentration (0.1%, 0.5% and 1% *w*/*w* in acetate buffer, pH 4.5). The reported transmittance values are the average of five consecutive measurements. All analyses were performed in triplicates.

#### 2.3.3. Formulation of the Hydrogel Delivery System

Hydrogels were prepared by dispersing under stirring at 60 °C the adequate amount of HPMC in acetate buffer pH 4.5 (for the control) and in CS or in CS nanoparticle dispersions (0.1% *w*/*w*, 0.5% *w*/*w* and 1% *w*/*w* in 200 mM acetate buffer, pH 4.5). Then, systems were cooled down under stirring and left at room temperature for 2 days to assure the complete hydration of HPMC before any further analysis. For the hydrogels containing nanoparticles, CS nanoparticle dispersions were prepared immediately before the addition of HPMC at final CS concentrations in the hydrogel of 0.1% *w*/*w*, 0.5% *w*/*w* and 1% *w*/*w*. The hydrogels were prepared using nanoparticles at certain CS/TPP ratios, selected according to DLS and transmittance analysis results. The ratios were 6:1 and 12:1 for the 0.5% *w*/*w* and 1% *w*/*w* chitosan nanoparticles, and 6:1 and 2:1 for the 0.1% *w*/*w* nanoparticles. For the preparation of the hydrogels loaded with metronidazole, the drug was dissolved in CS dispersions (free polymer or nanoparticles) at a concentration of 0.75% *w*/*w* (with respect to the final weight of the formulation), before adding HPMC.

#### 2.3.4. Rheological Analysis

Rheological analyses were performed using a stress-controlled rotational rheometer (Kinexus lab+, Malvern, UK) using a plate-cone geometry. The diameter of the cone was 40 cm and the angle was 4°. Samples, and the commercial formulation Zidoval^®^, used as a reference, were analysed by stress sweep and frequency sweep tests at 37 °C. Stress sweep analysis was performed at a frequency of 1 Hz in the range 0.05–20 Pa. For the frequency sweep, an increasing frequency in the range 0.01–1 Hz was applied to the samples at a constant stress (5 Pa). The measured rheological parameters were the elastic modulus (G’), the viscous modulus (G’’) and complex viscosity (Viscosity*). Analyses were performed in triplicates.

#### 2.3.5. Mucoadhesiveness Test

The mucoadhesiveness was evaluated *in vitro* by measuring the force required to detach the hydrogel from the contact with a mucin tablet, as reported in the literature [23]. Tablets were obtained by direct compression of mucin (400 mg) using a rotary tableting machine (Riva Piccola, Ronchi dei Legionari, Italy) equipped with punches of 11.28 mm and by applying a compression force of 27 KN (270 MPa) at 6 rpm. The obtained tablets had a hardness of 17 N and a thickness of 3.12 ± 0.05 mm. Tablet hardness was measured through a hardness tester (TBH30, Erweka, Heusenstamm, Germany). The thickness of the tablets was measured by a micrometer (103–137, Mitutoyo, Kawasaki, Japan). The mucoadhesive test was performed using a tensile tester apparatus (5543, Instron, Pianezza, Italy) equipped with a load cell of 5 N and a cylindrical probe with a diameter of 10 mm. The tablets were attached through a cyanoacrylate glue to the lower end of the cylindrical probe. Then, prior to the test, the tablets were hydrated by submersion in a 5% *w*/*w* of mucin dispersion in water for 5 min. The excess hydration liquid was removed from the tablet surface by gentle blotting. For testing, hydrogels were placed in glass vessels with diameters of 35 mm, and the probe was then lowered until the tablet was in contact with the surface of the hydrogel and a force of 0.01 N was recorded. The contact time was 2 min. After that, the cylindrical probe was moved up at a constant speed of 0.3 mm/s. The mucoadhesiveness of the hydrogels was expressed in terms of maximum mucoadhesive force (F_max_; the force required for the detachment of the mucin tablet from the surface of the hydrogel), displacement at the maximum force (S_max_), total work (Work_t_) and work at the maximum force (Work_In_). F_max_ and S_max_ were calculated from the peak value of the force vs. the displacement plot. Work_tot_ and Work_In_ were calculated from the total area and the partial area of the peak value below the plot force *vs* displacement, respectively. The reported values are the mean ± SD of five independent measurements.

#### 2.3.6. Franz Diffusion Cell Release Study

The release study was performed using a Franz-cell diffusion apparatus (Start-up 6-Cell Manual Test System, Hanson, Tulsa, OK, USA). Spectra/Por^®^ 1 Dialysis Membranes (MWCO 6000/8000 Da, pore size 0.45 μm, surface area 1 cm^2^) was placed between the donor and receptor chamber. The receptor chamber was filled with 6.5 mL of 200 mM acetate buffer pH 4.5 at 37 °C under constant stirring.

A quantity of 0.5 g of the analyzed hydrogels (HPMC 5.5%; HPMC 5.5%/CS 1%; HPMC 5.5% CS NPs 1% 6:1 and HPMC 5.5% CS NPs 1% 12:1) and 0.75% MTZ solution in 200 mM acetate buffer pH 4.5 (as reference) were placed onto the membrane under occlusion conditions. Samples from the receptor chamber (0.3 mL) were withdrawn at fixed time intervals (0, 15, 30, 60, 120, 240 min), and replaced with an equal volume of fresh acetate buffer. The amount of the released metronidazole was quantified by UV-spectroscopy (UV-1800 Shimadzu) through a calibration curve (λ = 277 nm, y = 0.01419x − 0.0071). The results were expressed as the mean ± SD cumulative release of metronidazole (%), with respect to the nominal amount of drug loaded in the formulation. Analyses were performed in triplicate.

#### 2.3.7. Antimicrobial Activity of the Hydrogels

The antimicrobial activity of the different hydrogels and the relative controls (chitosan dispersions) was tested using the agar well diffusion method (AWDM), in accordance with Campana et al. with slight modifications [24]. Briefly, several colonies were drawn from each plate of *Candida* spp., added to 30 mL of Triptone Soy Broth (TSB, Oxoid) and incubated at 37 °C for 24 h. At this point, each pathogen culture (about 107 CFU mL^−1^) was streaked on the surface of 25 mL of SDA plates and then kept at room temperature under flow cabinet for 20 min. Wells of 6 mm in diameter were made on the agar with sterile stainless-steel cylinders and 80 μL of each tested CS dispersion, and CS/HPMC hydrogels were pipetted into the wells. Fluconazole (Sigma) solutions (25 and 50 μg/mL) were also tested as controls. After 24 h of incubation at 37 °C, the diameter of the inhibition zone around each well was measured, and the antimicrobial activity was expressed as the mean ± standard deviation of the inhibition diameters produced by each sample. All the experiments were performed in duplicate.

#### 2.3.8. Statistical Analyses

Statistical analyses were performed with MINITAB^®^ Release 14.1 (Minitab Inc., Coventry, UK, 1972–2003) software. All statistical comparisons were made through a one-way ANOVA test with *p* values < 0.05 considered statistically significant.

## 3. Results and Discussions

### 3.1. Characterization of Chitosan Nanoparticles

**Figure 1:**
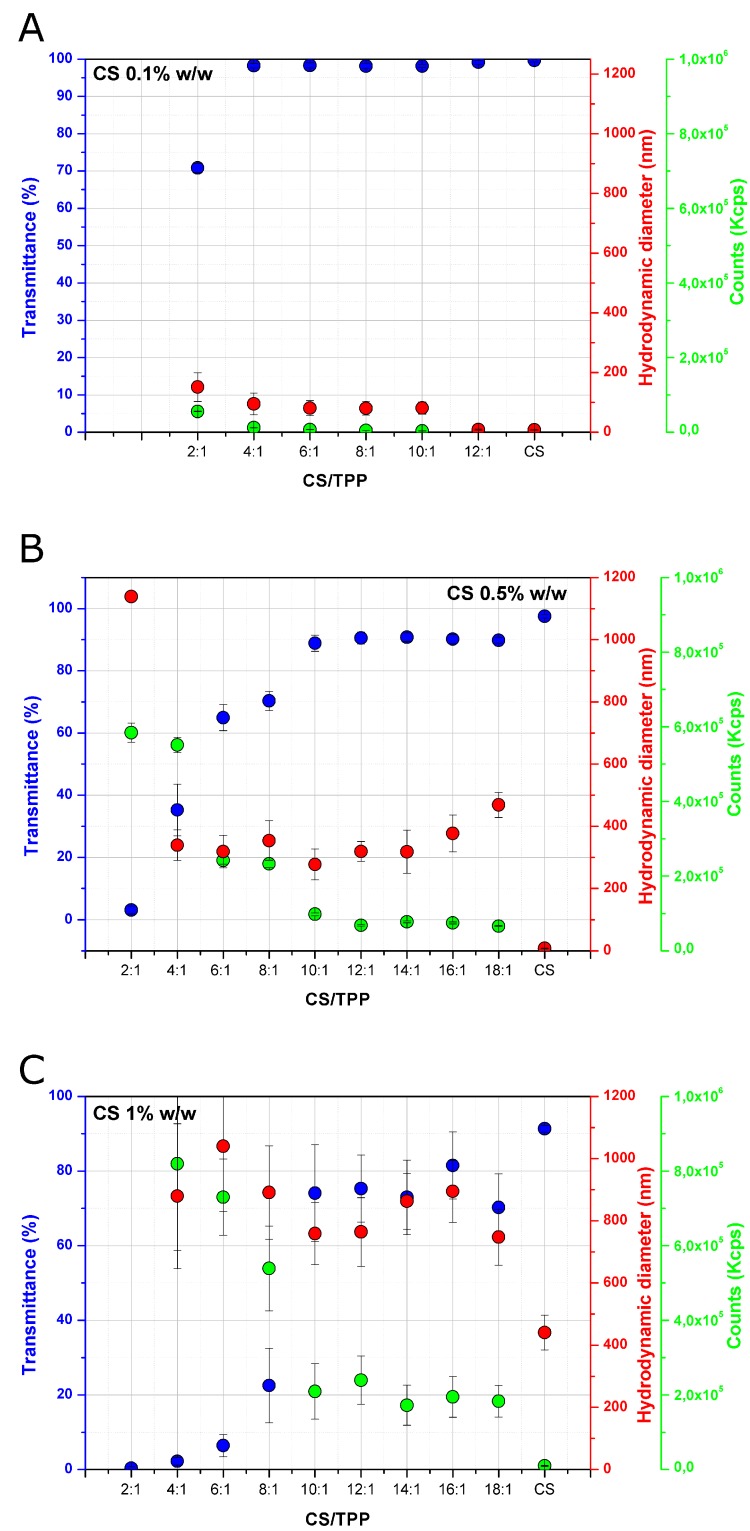
Hydrodynamic diameter (nm), counts (Kcps) and transmittance (%) of nanoparticles at different chitosan (CS) concentrations (0.1% (**A**), 0.5% (**B**) and 1% (**C**); *w*/*w*) and different CS/sodium tripolyphosphate (TPP) ratios prepared in 200 mM acetate buffer pH 4.5. Values are reported as mean ± SD of three independent measurements.

**Figure 2:**
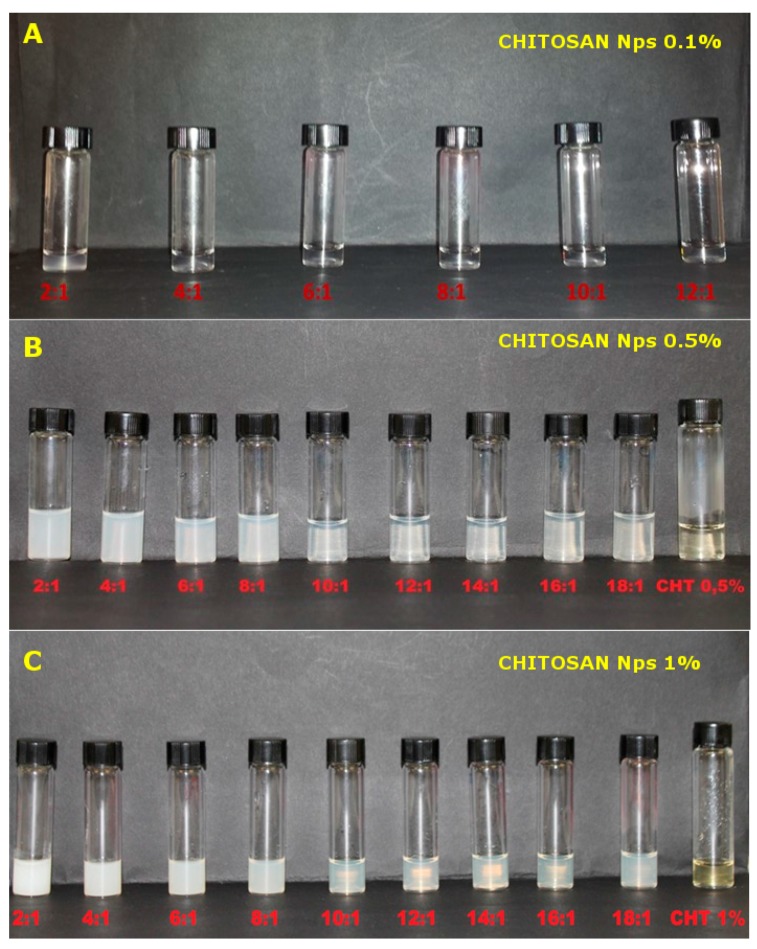
Image of the prepared chitosan nanoparticle dispersions in 200 mM acetate buffer pH 4.5 at the three different concentrations (0.1% (**A**), 0.5% (**B**) and 1% (**C**); *w*/*w*).

DLS measurements and transmittance analyses were performed for all the formulations, to investigate the effect of the chitosan concentration (%) and CS/TPP ratio on the formation and size of the obtained nanoparticles in acetate buffer, pH 4.5 (Figure 1). By comparing the hydrodynamic diameters calculated for the nanoparticles prepared at different CS concentrations, it became clear that the percentage of CS markedly influences the nanoparticle size. For nanoparticles obtained with 0.1% *w*/*w* of chitosan, the hydrodynamic diameter was found to be in the range of 85–95 nm, while sizes of approximately 300 and 900 nm were detected for particles prepared with 0.5% *w*/*w* and 1% *w*/*w* of chitosan, respectively. The increase in size as a function of the CS concentration is well documented in the literature for nanoparticles prepared by ionic gelation [25,26]. On the contrary, the effect of the CS/TPP ratio is less straightforward and it required a more detailed analysis. Indeed, the size evolution nanoparticles as a function of the CS/TPP ratio displayed a typical profile at any tested concentration. Nanoparticle size remained constant in a specific range of CS /TPP ratios in a chitosan concentration dependent manner. Specifically, the range of CS/TPP ratios in which the size of the nanoparticles was similar was found to be between 10:1 and 16:1 for 0.5% *w*/*w* and 1% *w*/*w* CS concentrations and between 4:1 and 10:1 for 0.1% *w*/*w* CS concentration. For ratios above this range, the formation of nanoparticles generally did not occur, while for lower ratios, a progressive increase in size was observed, leading to flocculation and CS precipitation at a ratio of 2:1 for 0.5% *w*/*w* and 1% *w*/*w* of chitosan and 1:1 for 0.1% *w*/*w* of chitosan. To evaluate the relevance of these two specific parameters—the CS/TPP ratio and chitosan concentration (%)—on nanoparticle size, a two-way variance analysis was performed. For this purpose, 18:1, 16:1 and 14:1 ratios were not included in the analysis because they did not produce any nanoparticles. In addition, the 2:1 ratio was also excluded since large aggregates were observed and they could affect the statistical analysis, leading to false positive results. All the considered parameters (CS concentration, CS/TPP ratio and the interaction term) were statistically significant (*p* value < 0.001 for CS concentration and CS/TPP ratio and 0.005 for the interaction term), but the CS concentration had a higher impact on the size (F value for CS concentration was at least 75 times higher than for the other two parameters). The effect of all the parameters considered can be easily displayed through the main effect and interaction plot (S1 and S2). A rise in CS concentration resulted in an increase of the nanoparticle size, while an opposite and limited effect was observed for CS/TPP ratios (main effect plot, Figure S1). Interestingly, the effect of CS/TPP ratio was much more pronounced at the highest CS concentration (interaction plot, Figure S2). Counts (Kpcs) were also recorded through DLS, as they represent a measure of the scattered light intensity received by the detector, which depends on the nature, size and concentration of the disperse system. At higher CS/TPP ratios (above 10:1 for nanoparticles containing 0.5% *w*/*w* and 1% *w*/*w* of chitosan and above 4:1 for systems containing 0.1% *w*/*w* chitosan), counts were constant, but at lower ratios, they progressively increased. Considering that the size of nanoparticles did not increase markedly over the different ratios (except for 2:1), it can be speculated that the increase in counts could be related to the presence of a higher number of nanoparticles at lower CS/TPP ratios. To validate this hypothesis, the transmittance was also measured. It can be observed that the increase in counts was accompanied by a decrease in the measured transmittance value of the samples. The variation in the optical properties of the samples at different CS/TPP ratios and chitosan concentrations (%) can be also seen by a visual observation of the samples. Indeed, systems at 0.1% *w*/*w* CS concentration are transparent (except for the 2:1), while at higher concentrations of chitosan (0.5%* w*/*w* and 1% *w*/*w*), as the CS/TPP ratio lowers, a gradual increase in the opacity of the samples can be observed (Figure 2).

In addition to pH 4.5, CS nanoparticles in 200 mM acetate buffer were also prepared at pH 5 and pH 5.5, to evaluate the effect of pH on nanoparticles size. Results from this investigation can be found in the supplementary materials (Figures S3 and S4).

### 3.2. Rheological Analysis of HPMC/CS Hydrogels

A preliminary screening was performed, in order to investigate the HPMC concentration (%) that is required to produce hydrogels in acetate buffer, pH 4.5, showing a consistency comparable to that of a jellified commercial formulation intended for vaginal administration. For comparison, the marketed hydrogel known as Zidoval^®^ was selected. Zidoval^®^ is formulated using a carboxypolymethylene polymer (Carbopol 974P), that displays carboxylic groups in the backbone. The polymeric dispersion thickens and forms a hydrogel after neutralization with inorganic (e.g., sodium hydroxide) or organic (e.g., triethanolamine) bases. At this pH value, chitosan precipitation occurs, therefore, carboxypolymethylene was not suitable for the preparation of mixed chitosan hydrogels. HPMC was chosen since the formation of the hydrogels is independent from the pH, due to the absence of ionisable groups.

**Figure 3:**
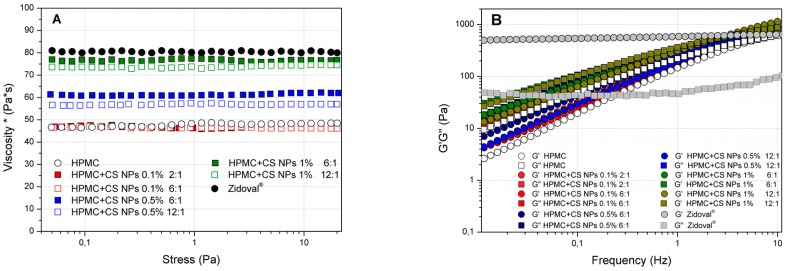
Rheological results of stress sweep (**A**) and frequency sweep (**B**) tests obtained from hydroxypropyl methylcellulose (HPMC)/Chitosan NPs hydrogels at 37 °C.

Hydrogel formulations prepared at 5.5% *w*/*w* HPMC concentration (control, CS and CS NPs 0.1% *w*/*w*, 0.5% *w*/*w* and 1% *w*/*w*) and Zidoval^®^ were compared by looking at the rheological parameter “complex viscosity” (viscosity*). Viscosity* is a measure of the viscosity of a semisolid system obtained through an oscillatory stress sweep test (Figure 3A). Only two CS/TPP ratios (6:1 and 2:1 for 0.1% *w*/*w* CS and 6:1 and 12:1 for 0.5% *w*/*w* and 1% *w*/*w* CS) were selected for the preparation of HPMC/CS NP hydrogels, in accordance with DLS and transmittance results. Hydrogels at the two CS/TPP ratios had a similar appearance to that of nanoparticle dispersions. Specifically, the hydrogels prepared at lower CS/TPP ratios were more opaque with respect to those prepared at the same concentration but at higher CS/TPP ratios (Figure S5).

In regard to the rheological results, 5.5% *w*/*w* HPMC hydrogel showed a viscosity* of 45 ± 3 Pa*s at 37 °C. The presence of chitosan, both as free polymer or as nanoparticles, increased the strength of the HPMC/chitosan mixed hydrogel. Particularly, a value of viscosity* comparable to Zidoval^®^ (75–80 Pa*s) was obtained at a concentration of 1% *w*/*w* chitosan, both as free polymer or nanoparticles (Figure 3A and Figure S6).

On the other side, the presence of chitosan did not markedly affect the viscoelastic properties of HPMC hydrogels, as we can see from the trend of the elastic (G’) or viscous (G’’) moduli (Figure 3B and Figure S6. However, what these analyses have pointed out is the different rheological behaviour of Zidoval^®^ with respect to HPMC hydrogels. Indeed, Zidoval^®^ can be considered a true gelling system from a rheological point of view. This is because the elastic modulus (G’) is higher than the viscous modulus (G’’) at any tested frequency (Figure 3B). In contrast, the prepared hydrogels cannot be defined as a gel from a rheological point of view, but they can be considered as concentrated polymer dispersions. In this case, the rheological moduli (G’ and G’’) are dependent on the applied frequency and show a frequency cross point. At frequencies below this point, the viscous modulus is higher than the elastic modulus and, consequently, they behave as a liquid dispersion. On the other side, at frequencies above the cross point, the elastic modulus is higher than the viscous modulus and they behave as weak gels. As such, the negligible effect of CS on the viscoelastic properties of 5.5% HPMC hydrogels can be evaluated in terms of cross points of the rheological moduli (G’ and G’’), which were found to be in the range 3–6 Hz for all the prepared systems (HPMC, CS/HPMC and CS NPs/HPMC hydrogels).

### 3.3. Mucoadhesiveness Test 

HPMC hydrogels containing 0.5% and 1% *w/w* of chitosan, both as free polymer and as nanoparticles, were analysed in term of their mucoadhesive properties. As shown in Table 1, a statistically significant increase (one-way ANOVA followed by Dunnett’s multiple comparisons test) in the mucoadhesiveness compared to the control (HPMC 5.5% *w/w* in acetate buffer 200 mM pH 4.5) was only observed for the hydrogels containing 1% *w/w* of chitosan (both as free polymer or as nanoparticles) by considering the maximum force (N) of detachment of the hydrated mucin tablet from the hydrogel surface (*p* < 0.007). A slight increase in the “total work” (mJ) and the “work at the maximum force” (mJ) were observed for the 1% *w/w* chitosan hydrogels, but in this case, such differences were not statistically significant with respect to the control. The results confirmed the mucoadhesive properties of HPMC. Indeed, at the concentration used, the mucoadhesion of the hydrogels is controlled by HPMC, while chitosan (as free polymer or nanoparticles) exerts a certain effect only at a concentration of 1% *w/w*. The effect on mucoadhesion of lower concentrations of chitosan (as 0.5% *w*/*w*) can be hindered by the presence of a larger amount of the mucoadhesive polymer, HPMC (5.5% *w*/*w*). This assumption is supported by the fact that in hydrogels formed by polymers with less mucoadhesive properties than HPMC (e.g., Poloxamer 407) [27], there is an effect of the presence of chitosan on mucoadhesion at a lower concentration [23].

### 3.4. Franz diffusion Cell In Vitro Release Study

**Figure 4:**
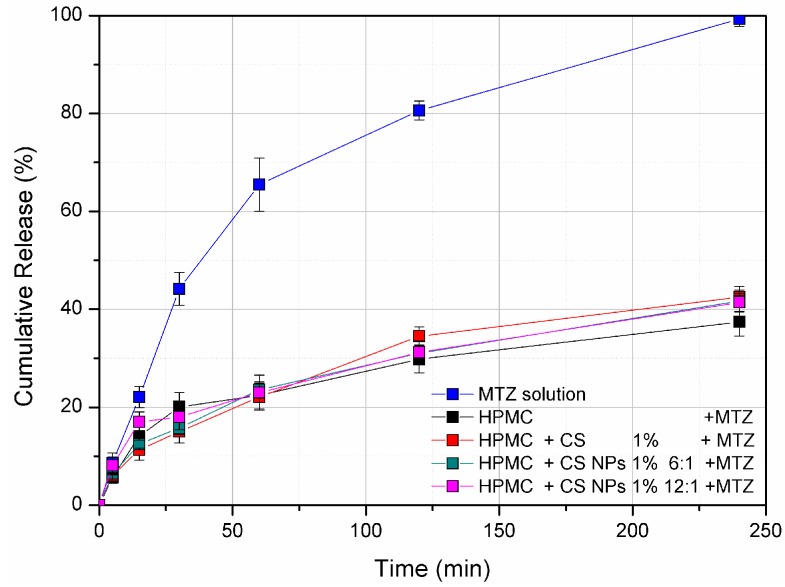
Metronidazole release from the prepared hydrogels (HPMC as control and HPMC/1% CS dispersion and HPMC/1% CS NPs) in 200 mM acetate buffer, pH 4.5, measured through Franz-diffusion cells apparatus. Values are reported as mean ± SD of three independent measurements.

Franz-cell diffusion studies were performed to investigate the influence of the presence of 1% *w*/*w* chitosan as free polymer or nanoparticles in the release of metronidazole from 5.5% *w*/*w* HPMC hydrogels. Only hydrogels containing 1% *w*/*w* of CS were analyzed, since they showed an effect on the mucoadhesive properties of the system. The calculated metronidazole cumulative release (%) over time from HPMC hydrogels and CS solution (as comparison) is shown in Figure 4. All the hydrogels displayed a similar release profile, independently of whether chitosan was present as a free polymer or as nanoparticles. This result is in accordance with the rheological analyses, which denoted the non-marked effect of the 1% *w*/*w* chitosan (as both free polymer and nanoparticles) on the viscoelastic properties of 5.5% *w*/*w* HPMC hydrogels. The release of metronidazole from these systems was found to be quite slow at the tested conditions (around 25–30% after 240 min) [28], while 100% release was achieved when the drug was dissolved in 200 mM acetate buffer.

### 3.5. Antimicrobial Activity of HPMC/CS Hydrogels

Several studies have been conducted to investigate the effect of chitosan or chitosan nanoparticles on fungal growth [29,30,31]. The obtained results have showed a marked antifungal activity exerted by chitosan against different fungal strains, including pathogenic species for humans as *Candida* spp. [20,32,33]. Fungicidal activity was demonstrated for chitosan, possibly due to membrane damage as a consequence of the interaction between protonated amino groups with negatively-charged cell surface proteins [34]. Despite the conspicuous number of experimental works, there is still a debate regarding the greater efficacy and broader activity of nanoparticles with respect to chitosan as free polymer.

*Candida albicans* is the main fungus responsible for vaginal yeast infections, although less frequently co-infection with other non-albicans Candida species (*Candida glabrata*, *Candida lusitaniae*) can occur [35]. The infections by species other than *Candida albicans* are reported to be more resistant to common antimycotic treatments (azole drugs; e.g., fluconazole) and responsible for the recurrence of the infection [36,37]. In this work, a panel of eight strains of *Candida* spp. (four albicans and four non-albicans) was tested, in order to investigate the anti-Candida activity of 1% chitosan as free polymer and nanoparticles, formulated both as aqueous dispersions or dispersed in a 5.5% HPMC hydrogel.

Initially, the susceptibility of the selected *Candida* spp. strains to fluconazole, as an antifungal azole drug model, was studied. Most of the strains were resistant to fluconazole at the two tested concentrations (25 μg/mL and 50 μg/mL), with the exception of *C. albicans* 360923, which showed inhibition diameters of 20 ± 1.25 and 28 ± 0.75 mm for fluconazole 25 μg/mL and 50 μg/mL, respectively (Table S1), comparable to those already reported in the literature [38]. 

In the present study, MIC values for chitosan were not determined since its efficacy against *Candida* spp. is well-known from the literature [32,39]. The tested chitosan concentration (1% *w*/*w*) was selected as the highest that produced nanoparticles at the investigated experimental conditions. The aim was to compare the anti-Candida activity of CS (as both free polymer and nanoparticles) when dispersed into HPMC hydrogels with that of CS dispersions prepared at the same concentrations. The results obtained from the agar well diffusion method are summarized in Table 2. The 1%* w*/*w* CS dispersion in 200 mM acetate buffer, pH 4.5, displayed good activity against all the tested *Candida* spp. strains, with inhibition growth diameters ranging from 12 ± 0.78 mm for *C. albicans* 18/01, to 13 ± 0.27 mm for *C. albicans* 4940 and *C. glabrata* 4955. One-percent CS nanoparticles in 200 mM acetate buffer, pH 4.5, have, instead, a different profile of activity. Indeed, 1% CS NPs were ineffective against all the tested *C. albicans* strains, while showing a comparable inhibition growth effect to 1% *w*/*w* CS dispersion (as free polymer), against the tested non-albicans strains. By considering nanoparticles at different CS/TPP ratios, the activity of nanoparticles prepared at the ratio 12:1 was slightly higher than those prepared at 6:1 for all tested non-albicans strains. This could be related, as already reported in the literature, to the smaller particle size obtained for the systems prepared using a 6:1 CS/TPP ratio when compared to those obtained at 12:1 ratio [19].

In regard to the HPMC-based hydrogel formulation containing 1% chitosan as free polymer, it was able to inhibit the growth of all tested *Candida* spp. with a variable degree of activity. Generally, a slightly lower effect with respect to 1% *w*/*w* CS dispersion was observed, which could be related to the slow diffusion capacity of CS in the hydrogel matrix if compared to a buffer solution. Interestingly, hydrogels formulated with 1% *w*/*w* CS NPs, in contrast to 1% *w*/*w* CS NPs as a dispersion (which was active only on non-albicans species), showed activity against all tested albicans and non-albicans strains. Indeed, the greatest zone of inhibition was obtained with the hydrogel HPMC/CSNPs 12:1 against *C. lusitaniae* 360804 (15 ± 0.57 mm), followed by *C. glabrata* 4955 (12 ± 0.57 mm) and *C. albicans* 18/1 (11 ± 0.25 mm), whilst lower zones of growth inhibition were observed with the hydrogel HPMC-CSNPs 6:1 (from 11 ± 0.57 mm for *C. albicans* 360923 to 8 ± 0.25 mm for *C. albicans* 4940). Despite CS NPs/HPMC hydrogels being active against all tested strains, a higher susceptibility was observed for non-albicans species and NPs prepared at 12:1 CS/TPP ratio. The different susceptibility among the tested strains could be explained by considering the structural differences of the cell wall among *Candida* spp. The cell wall of *C. albicans*, indeed, has more chitin and less adhesin molecules than that of *C. glabrata* [29,40]. Thus, the anti-Candida activity of chitosan was more pronounced against the strains with more chitin in the cell wall. Moreover, the presence of HPMC could facilitate the penetration of chitosan in the cell wall of the strain (i.d. *C. albicans*), justifying the broader range of the activity of the hydrogels containing chitosan, in comparison to CS dispersions, when analysed at the same concentration. Moreover, as for CS dispersions, the higher activity of the hydrogels prepared with CS NPs at 12:1 could be related to the smaller size.

Since common antifungal drugs are insoluble or slightly soluble in water, their formulation as hydrogels, using therapeutically-active concentrations of the drugs, is not feasible. Indeed, they are commercialized as cream for vaginal administration, instead of hydrogels. The formulation as hydrogels could be still possible using ethanol as a co-solvent to promote the solubilization of the drug. However, ethanol can act as a non-solvent for chitosan, thereby destabilizing the formulation [41]. For these reasons, we decided to prepare CS/HPMC hydrogels loaded with metronidazole, an antiprotozoal drug, approved for the treatment of bacterial vaginosis as a 0.75% *w*/*w* hydrogel. Metronidazole does not have intrinsic anti-Candida activity, but hydrogels containing metronidazole are available on the market for the treatment of bacterial vaginosis. A formulation of hydrogels loaded with metronidazole in the presence of chitosan could be a strategy to formulate a jellified system with improved antimicrobial activity against both vaginal bacteria and *Candida* spp. As such, metronidazole has been already formulated in other chitosan-based dosage forms, in order to address a combined effect on bacterial and yeast infections of the vaginal mucosa [5]. Metronidazole was not encapsulated inside CS nanoparticles, but it was solubilized inside the polymeric matrix, in order to formulate a hydrogel at the same drug concentration (0.75% *w*/*w*) as the commercial formulations. Metronidazole solution 0.75% (in 200 mM acetate buffer, pH 4.5) and 0.75% metronidazole HPMC hydrogel, tested as controls, did not show any anti-Candida activity against all tested strains. Moreover, the addition of 0.75% to 1% CS dispersion or 1% CS/HPMC hydrogels (as free polymer or nanoparticles) did not determine any increase in the antimicrobial activity against all the tested *Candida* spp. strains. These results confirmed that the presence of metronidazole did not exert any influence on the intrinsic anti-Candida effect of chitosan when both formulated as nanoparticles and dispersed into a polymeric hydrogel.

## 4. Conclusions

HPMC based hydrogels in the presence of different concentrations (0.1%, 0.5% and 1% *w*/*w*) of low molecular weight chitosan, either as free polymer or as nanoparticles, were successfully formulated at pH 4.5 and characterized in terms of rheological and mucoadhesive properties. Hydrogels containing 1% *w*/*w* chitosan showed a suitable viscosity (comparable to that of a commercial formulation) and an improved mucoadhesiveness in comparison to the control. These features make these hydrogels suitable for the vaginal administration and have been proposed here as a strategy to treat vaginal co-infections. Indeed, when incorporated in a 5.5% *w*/*w* HPMC matrix in mixed hydrogels, chitosan maintained its wide spectrum of anti-Candida activity against both albicans and non-albicans strains. In particular, no large differences in terms of intrinsic activity, evaluated from the inhibition growth diameter measured by agar well diffusion method, were observed between the hydrogels prepared with chitosan as free polymer or as nanoparticles. However, few differences were found in regard to the susceptibility of the different strains, with an observed higher effect on non-albicans versus albicans species.

Overall, this study reports, for the first time, the anti-Candida activity of chitosan as free polymer or nanoparticles, dispersed in a polymeric matrix forming a hydrogel. Moreover, the results suggest the possibility of using mixed hydrogels, composed of a HPMC matrix in which chitosan is dispersed, for the treatment or prevention of vaginal Candida co-infections. In this regard, the presence of chitosan can broaden the activity toward *Candida* spp. of hydrogel formulations containing the antimicrobial drug, metronidazole, commonly employed for the treatment of vaginal infections.

## Figures and Tables

**Table 1:** Mucoadhesiveness parameters (F_max_; S_max_; Work_tot_, Work_in_) calculated from the *in vitro* mucoadhesiveness test performed on the prepared HPMC 5.5% (*w*/*w*) hydrogels in presence of 0.5% (*w*/*w*) and 1% (*w*/*w*) chitosan, both as free polymer and as nanoparticles. The reported values are the mean ± SD of five replicates.

Formulations	F_max_ (N)	S_Max_ (mm)	Work_tot_(mJ)	Work_in_ (mJ)
**HPMC**	0.017 ± 0.001	1.615 ± 1.107	0.057 ± 0.009	0.017 ± 0.003
**HPMC + CS 0.5%**	0.018 ± 0.001	1.176 ± 0.183	0.060 ± 0.020	0.019 ± 0.003
**HPMC + CS 1%**	0.021 ± 0.002 *	1.224 ± 0.231	0.064 ± 0.025	0.020 ± 0.014
**HPMC + CS NPs 1% 12:1**	0.022 ± 0.002 *	1.056 ± 0.232	0.065 ± 0.020	0.019 ± 0.004
**HPMC + CS NPs 1% 6:1**	0.023 ± 0.002 *	0.888 ± 0.267	0.060 ± 0.022	0.019 ± 0.018
**HPMC + CS NPs 0.5% 12:1**	0.018 ± 0.002	1.389 ± 0.182	0.058 ± 0.027	0.018 ± 0.006
**HPMC + CS NPs 0.5% 6:1**	0.020 ± 0.003	0.899 ± 0.250	0.051 ± 0.018	0.017 ± 0.004

* hydrogels formulation statistically different (*p* < 0.05) from the control (HPMC 5.5%).

**Table 2:** Antimicrobial activity of HPMC/CS hydrogels and relative controls (CS dispersions), assessed by the agar well diffusion method (AWDM) against eight strains of *Candida* spp. obtained from vaginal swabs. The data represent the growth inhibition diameter (in mm) of each tested sample. Values are reported as the mean ± SD of two replicates.

Inhibition Growth Diameter (mm)								
Formulations	*Albicans* Strains				*Non-Albicans* Strains			
	*C. albicans* 11/01	*C. albicans* 18/01	*C. albicans* 4940	*C. albicans* 360923	*C. glabrat* 104/1	*C. glabrata* 104/22	*C. glabrata* 49/55	*C. lusitaniae* 360804
**CS 1%**	12 ± 0.6	12 ± 0.8	13 ± 0.3	13 ± 0.2	12 ± 0.6	13 ± 0.2	13 ± 0.3	13 ± 0.2
**CS NPs 1% 6:1**	0	0	0	0	9 ± 0.2	9 ± 0.3	9 ± 0.3	10 ± 0.1
**CS NPs 1% 12:1**	0	0	0	0	12 ± 0.8	11 ± 0.6	12 ± 0.7	13 ± 0.2
**HPMC**	0	0	0	0	0	0	0	0
**HPMC CS 1%**	12 ± 0.5	12 ± 0.6	12 ± 0.4	11 ± 0.3	12 ± 0.4	11 ± 0.4	12 ± 0.3	11 ± 0.2
**HPMC + CS NPs 1% 6:1**	9 ± 0.2	8 ± 0.3	8 ± 0.2	11 ± 0.6	9 ± 0.3	9 ± 0.2	10 ± 0.5	9 ± 0.1
**HPMC + CS NPs 1% 12:1**	9 ± 0.2	11 ± 0.3	8 ± 0.2	9 ± 0.2	10 ± 0.8	9 ± 0.2	12 ± 0.6	15 ± 0.6
**MTZ 0.75%**	0	0	0	0	0	0	0	0
**CS 1% MTZ 0.75%**	12 ± 0.6	12 ± 0.8	13 ± 0.2	12 ± 0.8	13 ± 0.3	14 ± 0.3	14 ± 0.3	13 ± 0.2
**CS NPs 1% 6:1 + MTZ 0.75%**	0	0	0	0	9 ± 0.4	9 ± 0.4	9 ± 0.2	11 ± 0.2
**CS NPs 1% 12:1 + MTZ 0.75%**	0	0	0	0	12 ± 0.6	11 ± 0.5	12 ± 0.6	12 ± 0.7
**HPMC + MTZ 0.75%**	0	0	0	0	0	0	0	0
**HPMC+CS 1% + MTZ 0.75%**	13 ± 0.5	12 ± 0.5	11 ± 0.4	11 ± 0.2	13 ± 0.4	10 ± 0.4	11 ± 0.2	11 ± 0.2
**HPMC + CS NPs 1% 6:1 + MTZ 0.75%**	9 ± 0.2	9 ± 0.3	8 ± 0.2	9 ± 0.2	9 ± 0.2	9 ± 0.2	9 ± 0.2	11 ± 0.3
**HPMC + CS NPs 1% 12:1 + MTZ 0.75%**	12 ± 0.7	11 ± 0.6	12 ± 1.0	10 ± 0.4	10 ± 0.6	11 ± 0.5	12 ± 1.0	11 ± 0.6

## Supplementary Materials

The following are available online at http://www.mdpi.com/1999-4923/10/1/23/s1, Figure S1: “Main effect” plot relative to the factors (CS % and CS/TPP ratio) influencing the size of nanoparticles, Figure S2: “Interactions plot” between the factors (CS% and CS/TPP ratio) influencing the size of nanoparticles, Figure S3: Hydrodynamic diameter (nm), counts (Kcps) and transmittance (%) of nanoparticles at different CS concentrations (0.1%, 0.5% and 1%; *w/w*) and different CST/TPP ratio prepared in acetate buffer pH 5, Figure S4: Hydrodynamic diameter (nm), counts (Kcps) and transmittance (%) of nanoparticles at different CS concentrations (0.1%, 0.5% and 1%; *w/w*) and different CST/TPP ratios prepared in acetate buffer pH 5.5, Figure S5: Image of the prepared HPMC/CS mixed hydrogels in 200 mM acetate buffer pH 4.5. From left to right: 5.5% HPMC (control); 5.5% HPMC/1%CS; 5.5% HPMC/1%CS NPs 12:1; 5.5% HPMC/1%CS NPs 6:1, Figure S6: Stress sweep (A) and frequency sweep (B) results obtained from HPMC/CS mixed hydrogels at 37°C. CS was dispersed into HPMC as free polymer, Table S1: Growth inhibition diameter (in mm) of Fluconazole (25 and 50 μg/mL) tested against the different *Candida* spp. strains. Values are reported as the mean ± SD of two replicates.

Supplementary File 1
