# Supplementary material for: Chitosan Loaded into a Hydrogel Delivery System as a Strategy to Treat Vaginal Co-Infection
Source: Pharmaceutics. 2018 Feb 3;10(1):23. doi: 10.3390/pharmaceutics10010023 (PMC5874836; doi:10.3390/pharmaceutics10010023)
Supplement: Supplementary File 1 [file pharmaceutics-10-00023-s001.pdf]

Supplementary Materials: Chitosan Loaded into a Hydrogel Delivery System as a Strategy to Treat Vaginal Co-Infection

Diego R. Perinelli, Raffaella Campana, Athanasios Skouras, Giulia Bonacucina, Marco Cespi, Francesca Mastrotto, Wally Baffone and Luca Casettari

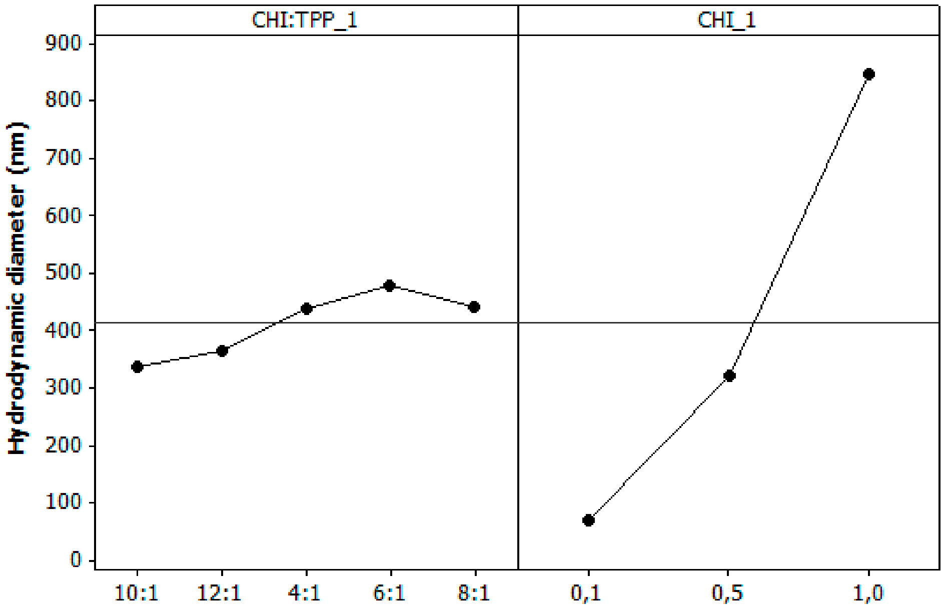

Figure S1. "Main effect" plot relative to the factors (CS % and CS/TPP ratio) influencing the size of nanoparticles.

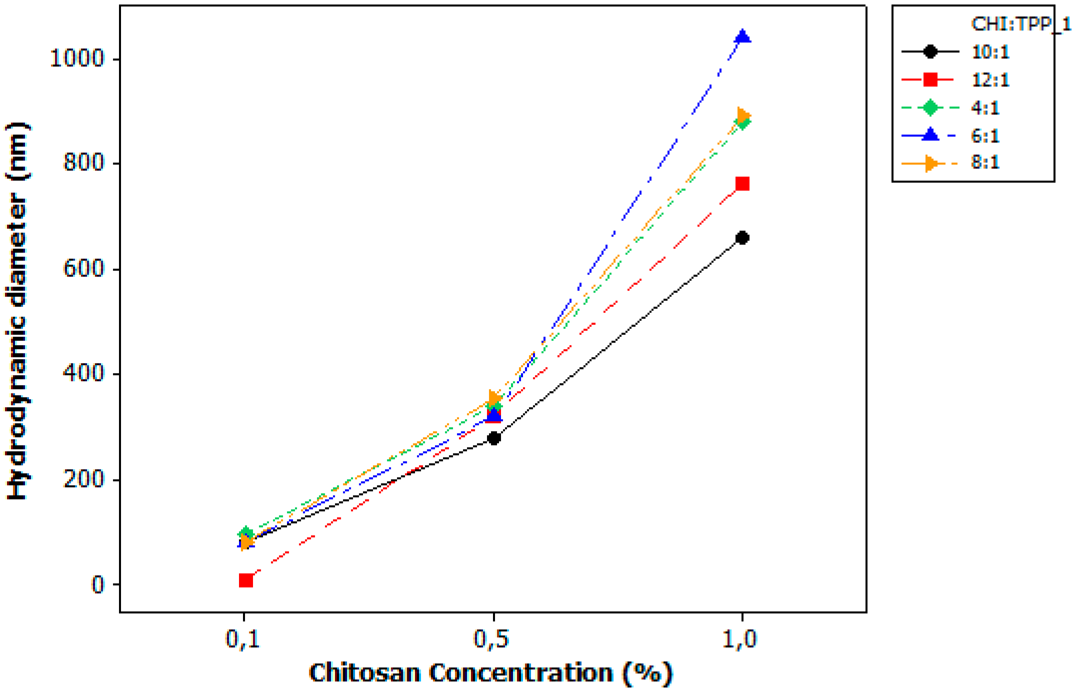

Figure S2. "Interactions plot" between the factors (CS % and CS/TPP ratio) influencing the size of nanoparticles.

Characterization of CS nanoparticles Prepared in 200 mM Acetate Buffer pH 5 and pH 5.5

The pH value of the buffer used for the preparation of CS nanoparticles has a strong effect on nanoparticles size. By analysing CS nanoparticles prepared at the same concentration, a progressive rise of size occurred by increasing the pH from 4.5 to 5 and 5.5. For chitosan 0.1% *w/w* nanoparticles, size increased from approximately 90 nm at pH 4.5 to around 200 nm at pH 5 and 300-400 nm at pH 5.5. For 0.5% CS nanoparticles, size increased from 300 nm at pH 4.5 to around 400-500 nm at pH 5 and 600-700 nm at pH 5.5. 1% CS nanoparticles were obtained only at pH 4.5. By increasing the pH to 5 and 5.5, colloidal systems with a size above 1000 nm were produced.

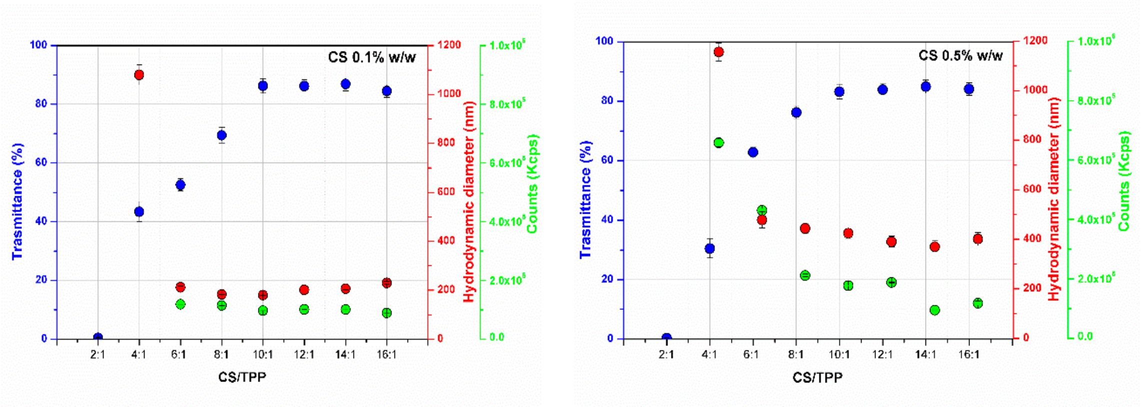

Figure S3. Hydrodynamic diameter (nm), counts (Kcps) and transmittance (%) of nanoparticles at different CS concentrations (0.1%, 0.5% and 1%; *w/w*) and different CS/TPP ratio prepared in acetate buffer pH 5.

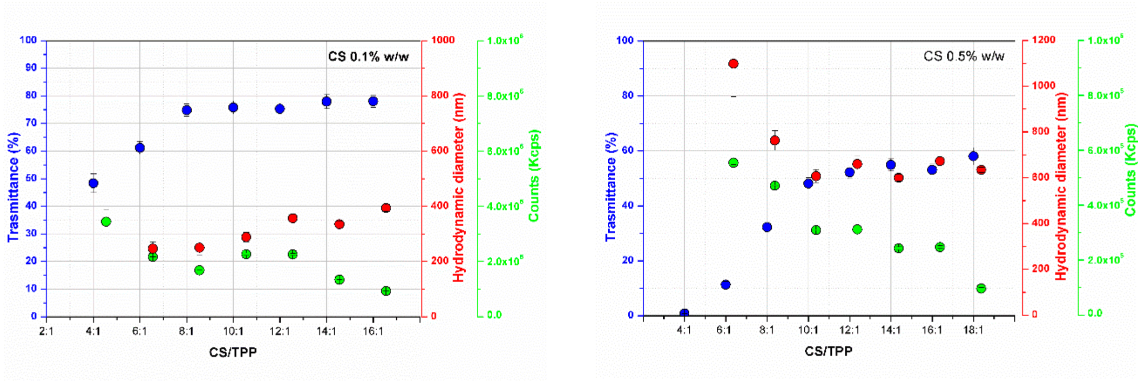

Figure S4. Hydrodynamic diameter (nm), counts (Kcps) and transmittance (%) of nanoparticles at different CS concentrations (0.1%, 0.5% and 1% *w/w*) and different CS/TPP ratios prepared in acetate buffer pH 5.5.

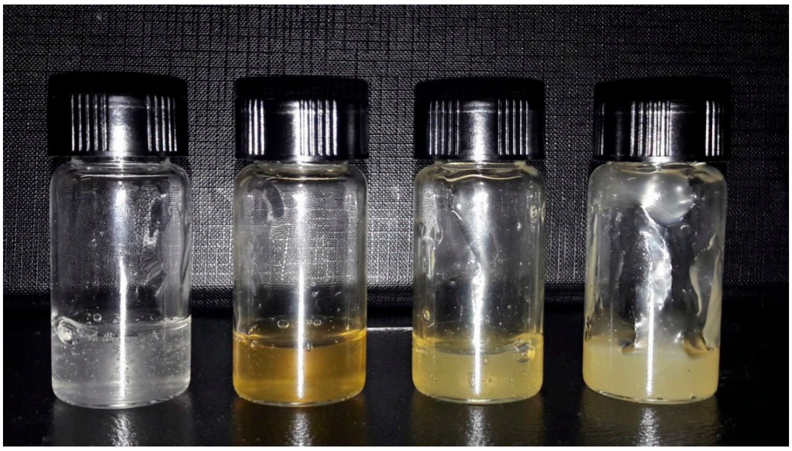

Figure S5. Image of the prepared HPMC/CS mixed hydrogels in 200 mM acetate buffer pH 4.5. From left to right: 5.5% HPMC (control); 5.5% HPMC/1%CS; 5.5% HPMC/1%CS NPs 12:1; 5.5% HPMC/1%CS NPs 6:1.

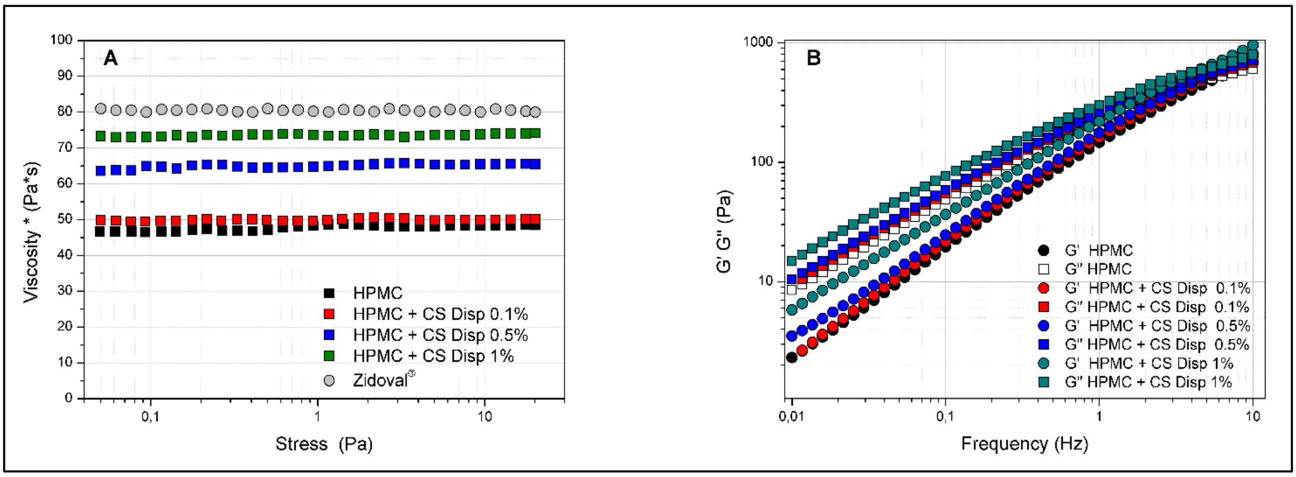

Figure S6. Stress sweep (**A**) and frequency sweep (**B**) results obtained from HPMC/CS mixed hydrogels at 37 °C. CS was dispersed into HPMC as a free polymer.

Table S1. Growth inhibition diameter (in mm) of Fluconazole (25 and 50 μg/mL) tested against the different *Candida* spp strains. Values are reported as the mean ± SD of two replicates.

**Antifungal**	**Inhibition growth diameter (mm)**							
	***albicans* strains**				***non-albicans* strains**			
	***C. albicans* 11/01**	***C. albicans* 18/01**	***C. albicans* 4940**	***C. albicans* 360923**	***C. glabrata* 104/1**	***C. glabrata* 104/22**	***C. glabrata* 49/55**	***C. lusitaniae* 360804**
**Fluconazole 25 μg/mL**	0	0	0	20±1.3	0	0	0	0
**Fluconazole 50 μg/mL**	0	0	0	28±0.8	0	0	0	0
